# The Effectiveness and Safety of Cinobufotalin Injection as an Adjunctive Treatment for Lung Cancer: A Meta-Analysis of Randomized Controlled Trials

**DOI:** 10.1155/2021/8852261

**Published:** 2021-02-05

**Authors:** Lin-Lu Li, Yi-Xin Su, Yun Mao, Peng-Yuan Jiang, Xue-Lei Chu, Peng Xue, Bo-Hui Jia, Shi-Jie Zhu

**Affiliations:** ^1^Graduate School, Beijing University of Chinese Medicine, Beijing 100029, China; ^2^Department of Oncology, Wangjing Hospital, China Academy of Chinese Medical Sciences, Beijing 100102, China

## Abstract

Cinobufotalin injection is a water-soluble preparation extracted from the skin secretion of *Bufo bufo gargarizans* Cantor or *B. melanotictus* Schneider, which has been widely used as an adjuvant treatment in lung cancer patients. This study aimed to evaluate the clinical efficacy and safety of cinobufotalin (PubChem CID: 259776) injection as an adjunctive treatment for lung cancer. We designed a meta-analysis that performed following the PRISMA (Preferred Reporting Items for Systematic Reviews and Meta-Analyses) guidelines. We aim to include randomized controlled trials by systematically searching the PubMed, EMBASE, CNKI, Wanfang database, VIP, CBM, the Cochrane Central Register of Controlled Trials, and Chinese Clinical Trial Registry from inception to Mar 1, 2020, comparing the difference between the use of cinobufotalin injection as an adjunctive treatment and a control group without cinobufotalin injection. The objective response rate (ORR) and quality of life (QOL) will be defined as the primary outcomes, and the disease control rate (DCR) and adverse events will be defined as the secondary outcomes. We included 21 articles with 1735 cases of lung cancer patients. Comparison results show that combining with cinobufotalin injection can improve ORR (OR = 1.77, 95% CI [1.43, 2.21], *P* < 0.001), with low heterogeneity (*P* = 0.94, *I*^2^ = 0%); DCR (OR = 2.20, 95% CI [1.70, 2.85], *P* < 0.001), with low heterogeneity (*P* = 0.60, *I*^2^ = 0%); KPS score (OR = 3.10, 95% CI [2.23, 4.32], *P* < 0.001), with low heterogeneity (*P* = 0.85, *I*^2^ = 0%); and the effect of pain relief (OR = 2.68, 95% CI [1.30, 5.55], *P* = 0.008), with low heterogeneity (*P* = 0.72, *I*^2^ = 0%). Low-to-moderate evidence shows that cinobufotalin injection combined with chemotherapy can significantly increase ORR, DCR, QOL, and the effect of pain relief. Meanwhile, cinobufotalin injection did not bring additional adverse events such as hematological toxicity, gastrointestinal toxicity, cardiotoxicity, hepatotoxicity, and nephrotoxicity; however, multicenter, large-sample, high-quality clinical research results are still needed to reveal the therapeutic effect of cinobufotalin injection in small-cell lung cancer (PROSPERO registration number: CRD42020170052).

## 1. Introduction

Lung cancer is one of the malignant neoplasms with a high global incidence, and its morbidity and mortality rates remain high [1, 2], including small-cell lung cancer (SCLC) and non-small-cell lung cancer (NSCLC). Data showed that the incidence of lung cancer in China was 36.71 per 100000 and the mortality rate was 28.49 per 100000 [3]. Cancer is the second leading cause of death in the United States [4], and in China, the most common cause of cancer-related death is lung cancer (26.4% of all cancers among men and 20.3% among women) [5]. The main treatments for lung cancer include chemotherapy, radiotherapy, surgery, targeted therapy, immunotherapy, and various clinical studies are ongoing research [6–11], which have brought a new revolution in the treatment of lung cancer but still limited. In some patients, underlying diseases may prevent clinical treatment from proceeding in full accordance with guideline recommendations, and these treatments may lead to unavoidable adverse events that may affect the quality of life of some patients [12, 13].

In recent years, the efficacy of Traditional Chinese Medicine (TCM) in treating diseases and its role in relieving discomfort have gradually been recognized. Chinese herbal medicine is an important part of TCM, including botanical medicines, animal medicines, and mineral medicines [14]. The common usages include Chinese herbal decoction, external acupoint application or lotion, Chinese patent medicine, etc. Cinobufotalin injection (Z34020273/Z34020274, China Food and Drug Administration) is a Chinese patent medicine extracted from the skin secretion of *Bufo bufo gargarizans* Cantor or *B. melanotictus* Schneider [15]. As a Chinese patent medicine made from animal medicine, it has been approved by the China Food and Drug Administration (ISO9002) for the treatment of malignant tumors [16]. Ma [17] compared the efficacy of CAP regimen and cinobufotalin injection in patients with lung cancer and found that patients in the cinobufotalin injection group had fewer gastrointestinal side effects, alopecia, myelosuppression, nephrotoxicity, and hepatotoxicity, but lower efficiency. A lower rate of pleural fluid control was also demonstrated in a study by Zhang et al. [18], which suggest that cinobufotalin injection can be more competent as an adjunctive treatment rather than as the primary treatment. Clinical trials have shown that the application of cinobufotalin injection as an adjuvant treatment for lung cancer can prolong patients' survival time and improve the quality of life [19, 20].

By searching the meta-analysis of lung cancer with the use of cinobufotalin injection, we found pieces of literature for non-small-cell lung cancer [21, 22]. Therefore, it is necessary to further evaluate the efficacy and safety of the cinobufotalin injection as an adjuvant therapy in lung cancer patients to provide an evidence-based basis for the use of the cinobufotalin injection in lung cancer.

## 2. Materials and Methods

This systematic review and meta-analysis was performed according to the Preferred Reporting Items for Systematic Reviews and Meta-Analyses guidelines (PRISMA guidelines). Ethical approval is not required, as this study involved published studies.

### 2.1. Search Strategies

Two reviewers (L-LL and Y-XS) independently retrieved all the related studies, and published papers were searched through the following databases from their inception to Mar 1, 2020: PubMed, EMBASE, China National Knowledge Infrastructure (CNKI), Wanfang database, Scientific Journal Database (VIP), and Chinese Biomedical Database (CBM). Unpublished materials were searched through the following databases from their inception to Mar 1, 2020: the Cochrane Central Register of Controlled Trials and Chinese Clinical Trial Registry. The search strategies in the electronic databases are listed in Table 1.

### 2.2. Inclusion and Exclusion Criteria

Two reviewers (L-LL and Y-XS) independently selected articles. Any disagreements or uncertainties were resolved by the third investigator (YM).

#### 2.2.1. Inclusion Criteria

Randomized controlled trials (RCTs) of cinobufotalin injection as the only adjuvant therapy for lung cancer were included. Patients diagnosed with lung cancer by histopathological or cytological diagnostic criteria were included. There were no restrictions on the treatment of the control group, that is, whether chemotherapy, radiation therapy, surgery, immunotherapy, or targeted therapy. The treatment in the experimental group consisted of cinobufotalin injection plus the therapy in the control group. There is no restriction of language, publication status, and literature types (journal articles, conference papers, or degree thesis).

#### 2.2.2. Exclusion Criteria

1. Animal research, cell experiments, reviews, retrospective studies, cohort studies, summary, case reports, commentaries, and noncontrol studies
2. Articles that are not degree thesis/dissertation but with only one author
3. Studies containing erroneous data and the author of the study cannot be contacted
4. Cinobufotalin injection is not the only adjuvant therapy; cinobufotalin injection is not used as adjuvant therapy and directly compared to antitumor therapies

### 2.3. Data Extraction

Two reviewers (L-LL and Y-XS) independently extracted data using a predesigned data extraction form. The third investigator compared the results to avoid bias in the data extraction process. Any disagreements or uncertainties were resolved by the third investigator (YM). Extracted details will include study characteristics (first author, year of publication, type of study), participants (age, gender), interventions (types of treatment), and results.

### 2.4. Outcome Definition

#### 2.4.1. Primary Outcomes

1. *Objective Response Rate (ORR).* ORR = CR + PR—complete response (CR), partial response (PR), stable disease (SD), and progressive disease (PD) [23]. Complete response (CR): the disappearance of all known disease not less than 4 weeks apart. Partial response (PR): 50% or more decrease in total tumor load of the lesions, not less than 4 weeks apart. No change (SD): a 50% decrease in total tumor size cannot be established, nor has a 25% increase in the size of one or more measurable lesions been demonstrated. Progressive disease (PD): 25% or more increase in the size of one or more measurable lesions or the appearance of new lesions.
2. *Quality of Life (QOL).* Patients' QOL will be evaluated by using Karnofsky Performance Status (KPS) [24] or Eastern Cooperative Oncology Group (ECOG) Performance Status [25].

#### 2.4.2. Secondary Outcomes

1. *Disease Control Rate (DCR).* DCR = CR + PR + SD.
2. *Adverse Events*. Adverse events include hematological toxicity; gastrointestinal toxicity; hepatic, renal, and cardiac injury; peripheral neurotoxicity; and alopecia, according to the WHO criteria [23] or the Common Terminology Criteria for Adverse Events [26].

### 2.5. Risk of Bias Assessment

Two reviewers (L-LL and P-YJ) independently assessed the risk of bias for each included study using the Cochrane Collaboration's risk of bias tool. The following items were evaluated: random sequence generation, allocation concealment, blinding of participants and personnel, blinding of outcome assessment, incomplete outcome data, selective outcome reporting, and other bias. Included studies will be classified as high, low, or unclear according to the type of bias and summarized in the bias risk table. Any disagreements or uncertainties were arbitrated by the third investigator (X-LC).

### 2.6. Statistical Analysis

Statistical analyses were carried out using Review Manager 5.3 software. Dichotomous variables were expressed as odds ratio (OR), and continuous variables were expressed as mean difference (MD). 95% CI was used for all data analyses. Heterogeneity between studies was assessed by Cochran's *Q* test. A random-effects model was used when heterogeneity was significant (*P* < 0.10, *I*^2^ > 50%), and a fixed-effects model was used when heterogeneity was acceptable (P > 0.10, *I*^2^ < 50%). Funnel plots were used to reveal the potential publication bias when studies were ten or more.

## 3. Results

### 3.1. Search and Screening

**Figure 1:**
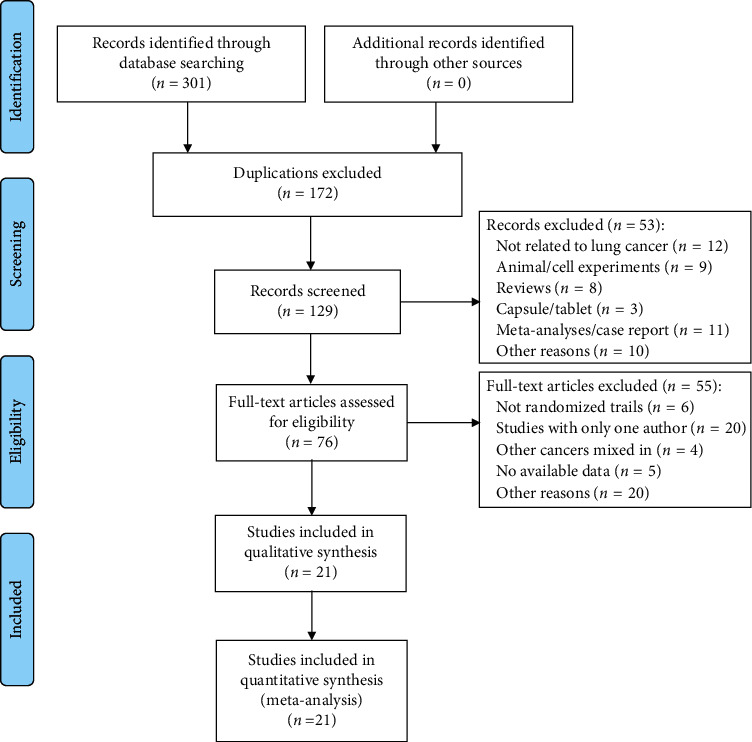
Flow diagram of the selection process.

A total of 295 papers and 6 trials were identified, of which 172 duplicate papers were excluded, 47 papers that did not meet the writing requirements were excluded based on their abstracts, and the 6 trials were not related to lung cancer. A complete literature review of 76 papers was performed, resulting in the inclusion of 21 papers [27–47], which included 1735 cases of lung cancer patients. The screening process is shown in Figure 1.

### 3.2. Characteristics of Included Studies

In the 21 papers included, the control group was treated with chemotherapy and the experimental group was treated with a combination of cinobufotalin injection and the same chemotherapy as the control group. Common chemotherapy regimens include docetaxel in combination with cisplatin (DP), etoposide in combination with cisplatin, gemcitabine in combination with cisplatin (GP), paclitaxel in combination with cisplatin (TP), vinorelbine in combination with cisplatin (NP), etc. The dose of the cinobufotalin injection is 10–30 mL/time/day with each cycle lasting 5, 7, 10, 14–15, 20–21, or 28 days, 1–6 cycles of treatment by intravenous injection. See Table 2.

### 3.3. Quality Assessment

**Figure 2:**
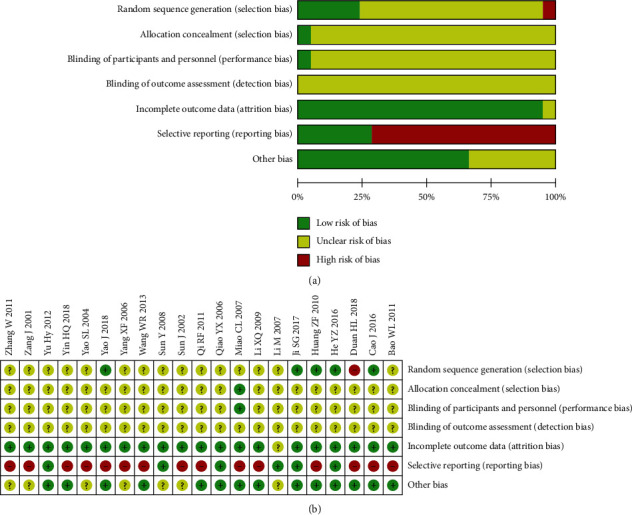
(a, b) Quality assessment.

The 21 studies included are shown in Figure 2 for quality assessment. 1 article randomized according to the order of hospitalization [29]. 5 studies used the random number table method [28, 30–32, 42]. 1 article applied the distribution concealment [35]. 1 article was double-blind [35]. The 21 studies did not report blind outcome assessments. 1 study mentioned that 22 patients were excluded due to inability to measure after surgery, missing data were unavailable, and there was no difference in the number of patients excluded between the two groups [33]. 15 studies reported a subset of splittable results and did not include a statement (e.g., only platelets, leukocytes, and no hemoglobin were reported) [27–29, 31, 34–36, 38, 40–44, 46, 47]. 7 studies did not mention baseline comparability [33, 38, 39, 41, 43, 46, 47].

### 3.4. Meta-Analysis Results

#### 3.4.1. Tumor Responses

**Figure 3:**
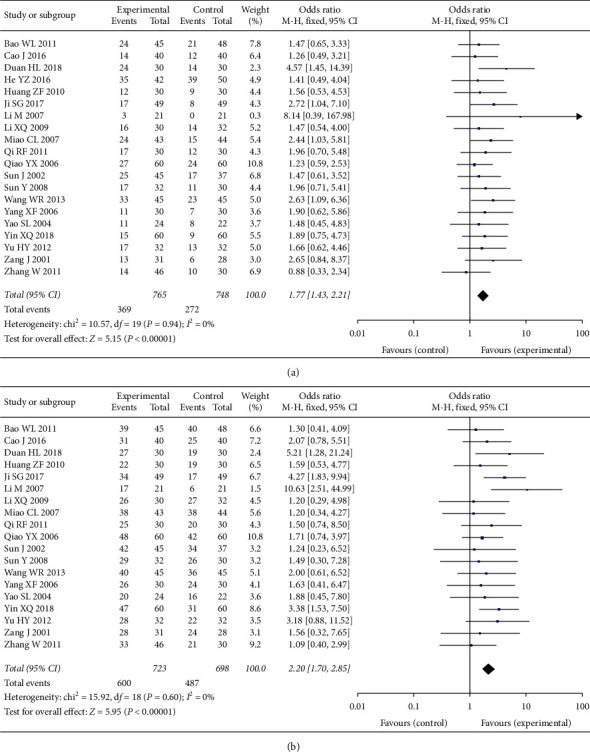
(a) Objective response rates in two groups. (b) Disease control rate in two groups.

Comparison of objective response rates was based on the pooled OR of 20 studies, with 765 cases in the experimental group and 748 cases in the control group. The pooled results indicated that the objective response rate in the experimental group can be improved (OR = 1.77, 95% CI [1.43, 2.21], *P* < 0.001), with low heterogeneity (*P* = 0.94, *I*^2^ = 0%); a fixed-effect model was used. See Figure 3(a)

Comparison of disease control rates was based on the pooled OR of 19 studies, with 723 cases in the experimental group and 698 cases in the control group. The pooled results indicated that the disease control rate in the experimental group can be improved (OR = 2.20, 95% CI [1.70, 2.85], *P* < 0.001), with low heterogeneity (*P* = 0.60, *I*^2^ = 0%); a fixed-effect model was used. See Figure 3(b).

#### 3.4.2. Quality of Life

**Figure 4:**
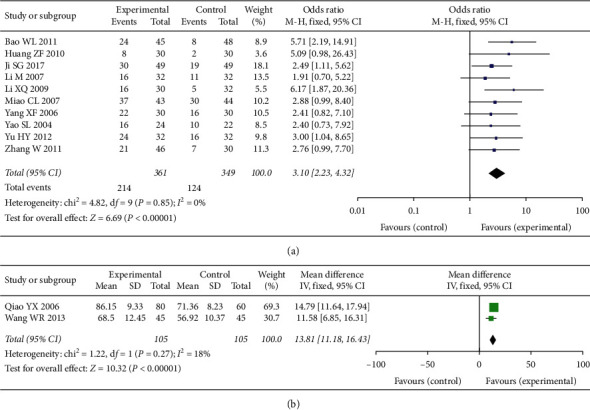
(a, b) KPS in two groups.

Comparison of KPS was based on the pooled OR of 10 studies, with 361 cases in the experimental group and 349 cases in the control group. The pooled results indicated that the quality of life in the experimental group can be improved (OR = 3.10, 95% CI [2.23, 4.32], *P* < 0.001), with low heterogeneity (*P* = 0.85, *I*^2^ = 0%); a fixed-effect model was used. See Figure 4(a).

The KPS of 2 studies are displayed as mean values and was analyzed statistically separately. Comparison of KPS scores was based on pooled SMD, with 105 cases in the experimental group and 105 cases in the control group. The pooled results indicated that the KPS score in the experimental group can be improved (MD = 13.81, 95% CI [11.18, 16.43], *P* < 0.001), with low heterogeneity (*P* = 0.27, *I*^2^ = 18%); a fixed-effect model was used. See Figure 4(b).

#### 3.4.3. Weight Change

**Figure 5:**
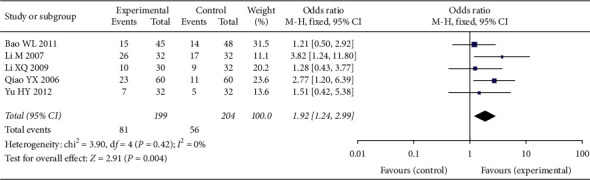
Weight change in two groups.

Comparison of weight based on the pooled OR of 5 studies, with 199 cases in the experimental group and 204 cases in the control group. The pooled results indicated that the body weight in the experimental group can be improved (OR = 1.92, 95% CI [1.24, 2.99], *P* < 0.001), with low heterogeneity (*P* = 0.42, *I*^2^ = 0%); a fixed-effect model was used. See Figure 5.

#### 3.4.4. Pain Relief

**Figure 6:**
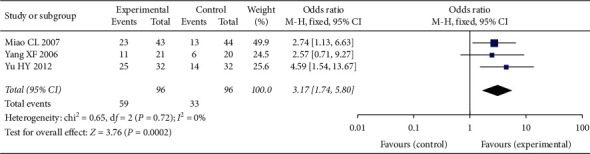
Pain relief in two groups.

Comparison of pain relief was based on the pooled OR of 3 studies, with 96 cases in the experimental group and 96 cases in the control group. The pooled results indicated that the pain relief in the experimental group can be improved (OR = 2.68, 95% CI [1.30, 5.55], *P* = 0.008), with low heterogeneity (*P* = 0.72, *I*^2^ = 0%); a fixed-effect model was used. See Figure 6.

#### 3.4.5. Adverse Events Assessment

**Figure 7:**
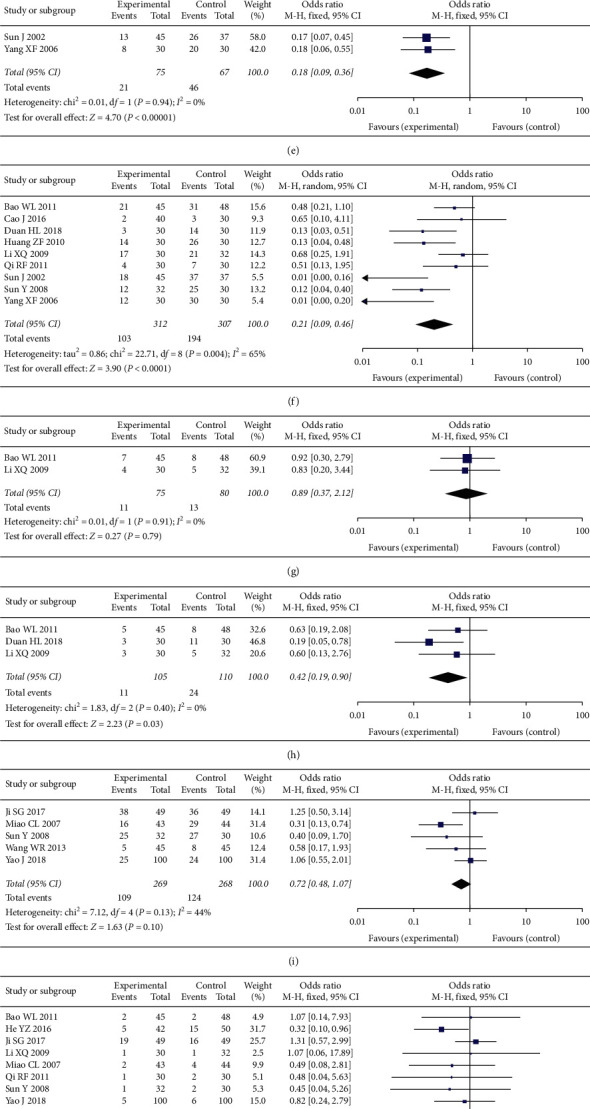
(a) Myelosuppression in two groups. (b) Leukopenia in two groups. (c) Hemoglobin in two groups. (d) Thrombocytopenia in two groups. (e) Neutropenia in two groups. (f) Nausea and vomiting in two groups. (g) Constipation in two groups. (h) Peripheral neurotoxicity in two groups. (i) Alopecia in two groups. (j) Hepatotoxicity in two groups. (k) Nephrotoxicity in two groups. (l) Cardiotoxicity in two groups. (m) Phlebitis in two groups. (n) Allergic in two groups.

Comparison of myelosuppression was based on the pooled OR of 3 studies, with 119 cases in the experimental group and 127 cases in the control group. The pooled results indicated that the occurrence of myelosuppression in the experimental group can be improved (OR = 0.36, 95% CI [0.18, 0.72], *P* < 0.001), with low heterogeneity (*P* = 0.63, *I*^2^ = 0%); a fixed-effect model was used. See Figure 7(a).

Comparison of leukopenia was based on the pooled OR of 14 studies, with 588 cases in the experimental group and 582 cases in the control group. The pooled results indicated that occurrence of leukopenia in the experimental group can be improved (OR = 0.34, 95% CI [0.19, 0.60], *P* < 0.001), with high heterogeneity (*P* < 0.001, *I*^2^ = 79%); a random-effect model was used. See Figure 7(b).

Comparison of hemoglobin reduction was based on the pooled OR of 3 studies, with 216 cases in the experimental group and 219 cases in the control group. The pooled results indicated that no significant difference in the occurrence of hemoglobin between the experimental group and the control group (OR = 0.74, 95% CI [0.37, 1.49], *P* = 0.4), with high heterogeneity (*P* = 0.02, *I*^2^ = 65%); a random-effect model was used. See Figure 7(c).

Comparison of thrombocytopenia was based on the pooled OR of 10 studies, with 381 cases in the experimental group and 376 cases in the control group. The pooled results indicated that occurrence of thrombocytopenia in the experimental group can be improved (OR = 0.44, 95% CI [0.25, 0.75], *P* = 0.003), with high heterogeneity (*P* = 0.01, *I*^2^ = 56%); a random-effect model was used. See Figure 7(d).

Comparison of neutropenia was based on the pooled OR of 2 studies, with 75 cases in the experimental group and 67 cases in the control group. The pooled results indicated that occurrence of neutropenia in the experimental group can be improved (OR = 0.18, 95% CI [0.09, 0.36], *P* < 0.001), with low heterogeneity (*P* = 0.94, *I*^2^ = 0%); a fixed-effect model was used. See Figure 7(e).

Comparison of nausea and vomiting was based on the pooled OR of 9 studies, with 312 cases in the experimental group and 307 cases in the control group. The pooled results indicated that the occurrence of nausea and vomiting in the experimental group can be improved (OR = 0.21, 95% CI [0.09, 0.46], *P* < 0.001), with high heterogeneity (*P* = 0.004, *I*^2^ = 65%); a random-effect model was used. See Figure 7(f).

Comparison of constipation was based on the pooled OR of 2 studies, with 75 cases in the experimental group and 80 cases in the control group. The pooled results indicated that no significant difference in the occurrence of constipation between experimental group and control group (OR = 0.89, 95% CI [0.37, 2.12], *P* = 0.79), with low heterogeneity (*P* = 0.91, *I*^2^ = 0%); a fixed-effect model was used. See Figure 7(g).

Comparison of peripheral neurotoxicity was based on the pooled OR of 3 studies, with 105 cases in the experimental group and 110 cases in the control group. The pooled results indicated that occurrence of peripheral neurotoxicity in the experimental group can be improved (OR = 0.42, 95% CI [0.19, 0.90], *P* = 0.03), with low heterogeneity (*P* = 0.40, *I*^2^ = 0%); a fixed-effect model was used. See Figure 7(h).

Comparison of alopecia was based on the pooled OR of 5 studies, with 269 cases in the experimental group and 3268 cases in the control group. The pooled results indicated that no significant difference in the occurrence of alopecia between the experimental group and the control group (OR = 0.72, 95% CI [0.48, 1.07], *P* = 0.10), with acceptable heterogeneity (*P* = 0.13, *I*^2^ = 44%); a fixed-effect model was used. See Figure 7(i).

Comparison of hepatotoxicity was based on the pooled OR of 8 studies, with 371 cases in the experimental group and 383 cases in the control group. The pooled results indicated that no significant difference in the occurrence of hepatotoxicity between the experimental group and the control group (OR = 0.73, 95% CI [0.45, 1.20], *P* = 0.22), with low heterogeneity (*P* = 0.69, *I*^2^ = 0%), a fixed-effect model was used. In Figure 7(j).

Comparison of nephrotoxicity was based on the pooled OR of 7 studies, with 329 cases in the experimental group and 333 cases in the control group. The pooled results indicated that no significant difference in the occurrence of nephrotoxicity between experimental group and control group (OR = 0.58, 95% CI [0.25, 1.38], *P* = 0.22), with low heterogeneity (*P* = 0.90, *I*^2^ = 0%); a fixed-effect model was used. See Figure 7(k).

Comparison of cardiotoxicity was based on the pooled OR of 2 studies, with 62 cases in the experimental group and 60 cases in the control group. The pooled results indicated that no significant difference in the occurrence of cardiotoxicity between experimental group and control group (OR = 0.64, 95% CI [0.10, 3.96], *P* = 0.63), with low heterogeneity (*P* = 0.73, *I*^2^ = 0%); a fixed-effect model was used. See Figure 7(l).

Comparison of phlebitis was based on the pooled OR of 4 studies, with 137 cases in the experimental group and 127 cases in the control group. The pooled results indicated that no significant difference in the occurrence of phlebitis between experimental group and control group (OR = 1.52, 95% CI [0.67, 3.43], *P* = 0.32), with low heterogeneity (*P* = 0.83, *I*^2^ = 0%); a fixed-effect model was used. See Figure 7(m).

Comparison of allergic was based on based on the pooled OR of 2 studies, with 94 cases in the experimental group and 94 cases in the control group. The pooled results indicated that no significant difference in the occurrence of allergic between the experimental group and the control group (OR = 1.00, 95% CI [0.31, 3.22], *P* = 1.00), with low heterogeneity (*P* = 0.55, *I*^2^ = 0%), a fixed-effect model was used. In Figure 7(n).

### 3.5. Publication Bias

**Figure 8:**
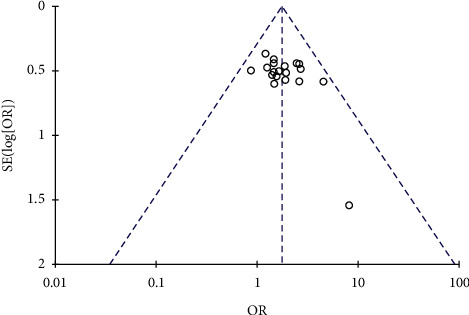
The publication bias analysis.

Funnel analysis of objective response rate as the primary outcome was performed. Since there are not more than 10 studies to evaluate the quality of life, no funnel diagram was drawn. The funnel plots of objective response rates is almost symmetrical, suggesting that there is little publication bias. See Figure 8.

## 4. Discussion

Lung cancer is one of the most common malignant tumors, accounting for 11.6 percent of the total 18.1 million new cancers in 2018 and had a higher mortality rate than other cancers, accounting for 18.4 percent of the 9.6 million deaths from cancer [1]; and lung cancer is also one of the most common cancer-related deaths in Europe [4], with an estimated 388,000 deaths [2]. In China, the incidence and mortality rate of lung cancer remain high [3, 5], and it is predicted that between 2015 and 2030, lung cancer mortality may increase by about 40% [3], but the five-year relative survival rate remains low, at 19.7% in 2012–2015 [48].

Due to complicated pathogenic factors, strong insidiousness of the disease, and adverse events of treatment, the combined treatment of Chinese and Western medicine for lung cancer has gradually gained attention in recent years and achieved certain effects. Studies have shown that Traditional Chinese medicine (TCM) can synergistically enhance the efficacy of chemotherapy and targeted therapy [49, 50]. We also found that TCM can also prolong survival time, prevent metastasis and relapse, and improve patients' quality of life in clinical practice. As a Chinese patent medicine approved for cancer, cinobufotalin injection has a significant clinical effect, which has also been confirmed in the literature. We designed this meta-analysis to explore the efficacy of cinobufotalin injection as adjuvant therapy for lung cancer.

In designing this meta-analysis, we did not specify which of the following treatments, including chemotherapy, radiation therapy, surgery, targeted therapy, and immunotherapy, were used as main therapy in our search; however, in addition to chemotherapy, other treatments combined with cinobufotalin injection to treat lung cancer have not been retrieved. After completing the search, we included RCTs in which cinobufotalin injection was the only adjunctive treatment versus a blank control. In addition to excluding studies with an erroneous primary outcome that is difficult to revise, we also excluded RCTs with only one author that were published in nonthesis/dissertation status as it is difficult for one person to implement an RCT. Two of the articles ultimately included were conference papers [31, 46], while the other 19 were journal articles. All the articles were retrieved from Chinese databases, and hence a potential of bias correction is required.

Most of the studies we included focused on non-small-cell lung cancer; only 1 study we retrieved included patients with small-cell lung cancer [43], and the data could not be utilized for subgroup analysis, suggesting that there is a considerable prospect for further exploration of the efficacy of small-cell lung cancer with cinobufotalin injection. 1 clinical study [20] with high quality was obtained through retrieval, and we did not include it to avoid confounding factors owing to the combination of other adjuvant therapies.

The results show that cinobufotalin injection does have the effect of assisting in enhancing the efficacy of chemotherapy, similar to existing studies. Studies have shown that the cinobufotalin injection may inhibit tumor growth by mediating the nonapoptotic death pathway of cyclophilin-D regulation in lung cancer cells [51]. Cinobufotalin injection can also increase the radiation sensitivity by inducing DNA fragmentation, thus slowing down and inhibiting DNA repair to produce an antitumor effect [52]. In our previous study, by pharmacological exploration, we have found that tumor growth can be inhibited by cell cycle inhibition and antiangiogenesis with the VEGFA epidermal growth factor receptor CASP3 AKT1 CCND1 associated with the prognosis of lung cancer patients [14]. A study by Zhang et al. [53] revealed that cinobufagin, which has a similar structure with cinobufotalin, can inhibit U2OS/MG-63 spheroid/mother cell survival in a time- and dose-dependent manner; and the tumorigenesis capability of osteosarcoma cells can be inhibited by blocking the IL-6-OPN-STAT3 signaling pathway.

For patients with advanced cancer, improved quality of life is also a great comfort to patients and their families. Our study results suggest that the use of cinobufotalin injection can improve the quality of life of lung cancer patients. Although 1 study [47] used a different rating scale, the removal/addition of this study did not affect this result.

The study by Jiang et al. [20] showed that patients with non-small-cell lung cancer treated with cinobufotalin injection had significantly better symptoms than the chemotherapy group, such as fatigue, nausea and vomiting, difficulty breathing, insomnia, loss of appetite, and diarrhea; and the cinobufotalin injection did not cause significant weight loss, bone marrow suppression, gastrointestinal reactions, hepatotoxicity, or nephrotoxicity. Few adverse reactions have been reported for Cinobufotalin injection, suggesting that the clinical application of Cinobufotalin injection has a good safety profile. Meng et al. [15] revealed that drug-related toxicity caused by the clinical study of the Huachansu injection was determined to be quite mild, with 73% of patients had no drug-related toxicity greater than grade I.

It is noteworthy that the pain relief effect of cinobufotalin injection is very significant, as we confirmed in our clinical application. Although there are only 3 studies [35, 41, 45] with combinable data, the heterogeneity is low. Cinobufagin has been found to alleviate cancer-related pain by promoting the enrichment of CD^3/4/8^ lymphocytes to tumors and adjacent tissues, activating the pro-papaverine/*β*-endorphin/*μ*-opioid receptor pathway, which has a similar chemical composition of cinobufotalin [54].

The meta-analysis revealed some irregularities in the papers included and the limitations of our study, which were listed below:

1. Some studies were more ambiguously described regarding design and implementation, suggesting that researchers may not have a thorough understanding of randomized controlled trials, which may result in a lower level of evidence for this meta-analysis.
2. Fewer clinical registries are searchable and the design of the trial is not available to us, which results in a difficulty of quality assessment of the literature and data extraction. It also raises the possibility that there is an irrational clinical study design and that clinical data can be withheld.
3. Only three of the included studies enrolled more than 100 patients. To improve the results of cinobufotalin injection, integration of multiple multicenter, large-sample clinical trials is still needed.
4. Many studies do not yet have standardized evaluation criteria and recording methods, such as myelosuppression scores, degree of symptom improvement, and the extent of body weight changes. This may allow the authors to report results tendentiously and cause some difficulties in data extraction and consolidation.
5. Some papers presented low-level errors, such as the sum of the events over the total number of this group. The authors do not have a thorough understanding of statistics, and in some papers, it can be found that the authors cannot discriminate the dichotomous variables and continuous variables. These errors may confuse other readers and mislead them to make errors in their next research.
6. By removing studies one by one, we found that a few studies were more likely to lead to heterogeneous in some types of outcomes. This suggests that such sources of publications are likely to result in less credible results and may provide other researchers with an erroneous guide.

## 5. Conclusion

In this meta-analysis, we found evidence that cinobufotalin injection combined with chemotherapy for non-small-cell lung cancer improves objective response rates, disease control, and quality of life with a good safety profile. However, unlike our original design, the results of our study still lack evidence to support the clinical efficacy of cinobufotalin injection in combination with other treatments (chemotherapy, radiotherapy, immunotherapy, targeted therapy); and it is hard to demonstrate the efficacy of cinobufotalin injection in patients with small-cell lung cancer. Higher-quality RCTs with larger sample sizes are needed to evaluate the efficacy of cinobufotalin injection in lung cancer.

## Figures and Tables

**Table 1:** The search strategies.

Search strategy of English database	
#1	“Lung Neoplasms” [Mesh]
#2	(((((((((((((((((Pulmonary Neoplasms [Title/Abstract]) OR Neoplasms, Lung [Title/Abstract]) OR Lung Neoplasm [Title/Abstract]) OR Neoplasm, Lung [Title/Abstract]) OR Neoplasms, Pulmonary [Title/Abstract]) OR Neoplasm, Pulmonary [Title/Abstract]) OR Pulmonary Neoplasm [Title/Abstract]) OR Lung Cancer[Title/Abstract]) OR Cancer, Lung [Title/Abstract]) OR Cancers, Lung [Title/Abstract]) OR Lung Cancers [Title/Abstract]) OR Pulmonary Cancer [Title/Abstract]) OR Cancer, Pulmonary [Title/Abstract]) OR Cancers, Pulmonary [Title/Abstract]) OR Pulmonary Cancers [Title/Abstract]) OR Cancer of the Lung [Title/Abstract]) OR Cancer of Lung[Title/Abstract]) OR ((((((((((((((((lung neoplasm [Title/Abstract]) OR lung carcinoma [Title/Abstract]) OR lung tumor [Title/Abstract]) OR lung malignant [Title/Abstract]) OR non-small cell lung neoplasm [Title/Abstract]) OR non-small cell lung carcinoma[Title/Abstract]) OR non-small cell lung tumor [Title/Abstract]) OR non-small cell lung cancer [Title/Abstract]) OR non-small cell lung malignant [Title/Abstract]) OR NSCLC [Title/Abstract]) OR small cell lung neoplasm [Title/Abstract]) OR small cell lung carcinoma [Title/Abstract]) OR small cell lung tumor [Title/Abstract]) OR small cell lung cancer [Title/Abstract]) OR small cell lung malignant [Title/Abstract]) OR SCLC [Title/Abstract])
#3	#1 OR #2
#4	(((cinobufotalin [Title/Abstract]) OR cinobufacini [Title/Abstract]) OR cinobufagin [Title/Abstract]) OR huachansu [Title/Abstract]
#5	Injection [Title/Abstract]
#6	(((((((((random∗ controlled trial [Title/Abstract]) OR random∗ controlled [Title/Abstract]) OR clinical trial [Title/Abstract]) OR RCT [Title/Abstract]) OR random∗[Title/Abstract]) OR controlled [Title/Abstract]) OR groups [Title/Abstract]) OR trial [Title/Abstract]) OR placebo [Title/Abstract]) OR clinical [Title/Abstract]
#7	#3 AND #4 AND #5 AND #6

Search strategy of Chinese database	
#1	“fei ai” OR “fei *e* xing zhong liu” OR “fei bu zhong liu” OR “fei bu zhong kuai” OR “xiong bu zhong liu” OR “xiong bu zhong kuai” OR “xiao xi bao fei ai” OR “fei xiao xi bao fei ai” OR “yuan fa xing zhi qi guan ai” OR “zhi qi guan ai” [Subject]
#2	“hua chan su” [Subject]
#3	“zhu she ye” OR “zhu she ji” [Subject]
#4	“sui ji dui zhao” OR “sui ji” OR “dui zhao” OR “dui zhao zu” OR “zhi yan zu” OR “zhi liao zu” OR “fen zu” OR “fen wei” OR “an wei ji” OR “lin chuang” OR “RCT” [Subject]
#5	#1 AND #2 AND #3 AND #4

**Table 2:** Characteristics of included studies.

Study	Enrollment period	Sample size		Intervention		Evaluation index
		Experimental group	Control group	Experimental group	Control group	
Bao WL 2011	2007.8–2010.10	45	48	GP + cinobufacini injection	GP	Adverse event, clinical efficacy, KPS, weight change
Cao J 2016	2013.1–2015.1	40	40	DP + cinobufacini injection	DP	Adverse event, clinical efficacy, median survival time, survival rate
Duan HL 2018	2015.1–2017.1	30	30	Docetaxel + cinobufacini injection	Docetaxel	Adverse event, clinical efficacy, tumor marker
He YZ 2016	2013.1–2015.1	42	50	TP + cinobufacini injection	TP	Adverse event, clinical efficacy, tumor marker
Huang ZF 2010	2006.8–2009.8	30	30	GC + cinobufacini injection	GC	Adverse event, clinical efficacy, Immunity, KPS, Zhengzhou score
Ji SG 2017	2014.6–2016.12	49	49	DC + cinobufacini injection	DC	Adverse event, clinical efficacy, KPS, mPFS
Li M 2007	2002.6–2006.6	32	32	NP/GP + cinobufacini injection	NP/GP	Clinical efficacy, KPS, Mean survival time, survival rate, symptoms, weight change
Li XQ 2009	2005.8–2007.10	30	32	NP + cinobufacini injection	NP	Adverse event, clinical efficacy, KPS, weight change
Miao CL 2007	2002.6–2005.2	43	44	NP + cinobufacini injection	NP	Adverse event, clinical efficacy, KPS, median response duration, median survival time, pain relief
Qi RF 2011	2008.6–2010.6	30	30	GP/NP/TP + cinobufacini injection	GP/NP/TP	Adverse event, clinical efficacy
Qiao YX 2006	1999.1–2004.1	60	60	NP + cinobufacini injection	NP	Adverse event, clinical efficacy, Immunity, KPS, survival rate, weight change
Sun J 2002	1998.2–2000.12	45	37	VP + cinobufacini injection	VP	Adverse event, clinical efficacy, KPS
Sun Y 2008	2003.5–2005.2	32	30	NI + cinobufacini injection	NI	Adverse event, clinical efficacy, KPS
Wang WR 2013	2010.6–2011.12	45	45	TP + cinobufacini injection	TP	Adverse event, clinical efficacy, KPS, QLQ-C30 score, tumor marker
Yang XF 2006	2003.8–2005.8	30	30	NP + cinobufacini injection	NP	Adverse event, clinical efficacy, KPS, pain relief
Yao J 2018	2013.1–2017.1	100	100	DP + cinobufacini injection	DP	Adverse event, immunity, pain relief, QLQ-C30 score, survival rate, Zhengzhou score
Yao SL 2004	2000.2–2004.2	24	22	CAP/EP + cinobufacini injection	CAP/EP	Clinical efficacy, KPS, white blood cell
Yin XQ 2018	2013.1–2016.12	60	60	EP + cinobufacini injection	EP	Adverse event, clinical efficacy
Yu HY 2012	2009.6.1–2010.12.31	32	32	DP + cinobufacini injection	DP	Adverse event, clinical efficacy, KPS, median survival time, pain relief, weight change
Zang J 2001	NG	31	28	NG + cinobufacini injection	NG	Adverse event, clinical efficacy,
Zhang W 2011	2009.12–2010.12	46	30	Docetaxel + cinobufacini injection	Docetaxel	Adverse event, clinical efficacy, ECOG score

## Data Availability

The data used to support the findings of this study are included within the supplementary information files.

## Abbreviations

CAP: Cyclophosphamide + Epirubicin + DDP
CBM: Chinese Biomedical Database
CI: Confidence interval
CNKI: China National Knowledge Infrastructure
CR: Complete response
DC: Docetaxel + carboplatin
DCR: Disease control rate
DDP: Cisplatin
DNA: Deoxyribonucleic acid
DP: Docetaxel + DDP
ECOG: Eastern Cooperative Oncology Group
EP: Etoposide + DDP
GCb: Gemcitabine + Carboplatin
GP: Gemcitabine + DDP
KPS: Karnofsky performance score
MD: Mean difference
mPFS: Median progression-free survival
NG: Not given
NI: Navelbine + Ifosfamide
NP: Navelbine + DDP
NSCLC: Non-small-cell lung cancer
OR: Odds ratio
ORR: Objective response rate
PD: Progressive disease
PR: Partial response
PRISMA: Preferred Reporting Items for Systematic Reviews and Meta-Analyses
QOL: Quality of life
RCT: Randomized controlled trial
SCLC: Small-cell lung cancer
SD: Stable disease
TCM: Traditional Chinese medicine
TP: Paclitaxel + DDP
VIP: Chinese Scientific Journal Database.

## Supplementary Materials

Supplementary Materials Tables S1 and S2 show the effectiveness of cinobufotalin injection. Tables S3 and S4 are about the KPS in two groups. Table S5 is a description of the weight change influenced by cinobufotalin injection. Table S6 shows the pain relief in two groups. Tables S7-S20 records the occurrence of adverse events in two groups.
