# Supplementary material for: The Effectiveness and Safety of Cinobufotalin Injection as an Adjunctive Treatment for Lung Cancer: A Meta-Analysis of Randomized Controlled Trials
Source: Evid Based Complement Alternat Med. 2021 Feb 5;2021:8852261. doi: 10.1155/2021/8852261 (PMC7881937; doi:10.1155/2021/8852261)
Supplement: Supplementary Materials — Tables S1 and S2 show the effectiveness of cinobufotalin injection. Tables S3 and S4 are about the KPS in two groups. Table S5 is a description of the weight change influenced by cinobufotalin injection. Table S6 shows the pain relief in two groups. Tables S7-S20 records the occurrence of adverse events in two groups. [file 8852261.f1.doc]

**Table S1 Objective response rates in two groups**

| Studies | Experimental | | Control | |
| --- | --- | --- | --- | --- |
|  | Events | Total | Events | Total |
| Duan HL 2018 | 24 | 30 | 14 | 30 |
| He YZ 2016 | 35 | 42 | 39 | 50 |
| Huang ZF 2010 | 12 | 30 | 9 | 30 |
| Ji SG 2017 | 17 | 49 | 8 | 49 |
| Li M 2007 | 3 | 21 | 0 | 21 |
| Li XQ 2009 | 16 | 30 | 14 | 32 |
| Miao CL 2007 | 24 | 43 | 15 | 44 |
| Qi RF 2011 | 17 | 30 | 12 | 30 |
| Qiao YX 2006 | 27 | 60 | 24 | 60 |
| Sun J 2002 | 25 | 45 | 17 | 37 |
| Sun Y 2008 | 17 | 32 | 11 | 30 |
| Wang WR 2013 | 33 | 45 | 23 | 45 |
| Yang XF 2006 | 11 | 30 | 7 | 30 |
| Yao SL 2004 | 11 | 24 | 8 | 22 |
| Yin XQ 2018 | 15 | 60 | 9 | 60 |
| Yu HY 2012 | 17 | 32 | 13 | 32 |
| Zang J 2001 | 13 | 31 | 6 | 28 |
| Zhang W 2011 | 14 | 46 | 10 | 30 |

**Table S2 Disease control rate in two groups**

| Studies | Experimental | | Control | |
| --- | --- | --- | --- | --- |
|  | Events | Total | Events | Total |
| Bao WL 2011 | 39 | 45 | 40 | 48 |
| Cao J 2016 | 31 | 40 | 25 | 40 |
| Duan HL 2018 | 27 | 30 | 19 | 30 |
| Huang ZF 2010 | 22 | 30 | 19 | 30 |
| Ji SG 2017 | 34 | 49 | 17 | 49 |
| Li M 2007 | 17 | 21 | 6 | 21 |
| Li XQ 2009 | 26 | 30 | 27 | 32 |
| Miao CL 2007 | 38 | 43 | 38 | 44 |
| Qi RF 2011 | 25 | 30 | 20 | 30 |
| Qiao YX 2006 | 48 | 60 | 42 | 60 |
| Sun J 2002 | 42 | 45 | 34 | 37 |
| Sun Y 2008 | 29 | 32 | 26 | 30 |
| Wang WR 2013 | 40 | 45 | 36 | 45 |
| Yang XF 2006 | 26 | 30 | 24 | 30 |
| Yao SL 2004 | 20 | 24 | 16 | 22 |
| Yin XQ 2018 | 47 | 60 | 31 | 60 |
| Yu HY 2012 | 28 | 32 | 22 | 32 |
| Zang J 2001 | 28 | 31 | 24 | 28 |
| Zhang W 2011 | 33 | 46 | 21 | 30 |

**Table S3 KPS in two groups**

| Studies | Experimental | | Control | |
| --- | --- | --- | --- | --- |
|  | Events | Total | Events | Total |
| Bao WL 2011 | 24 | 45 | 8 | 48 |
| Huang ZF 2010 | 8 | 30 | 2 | 30 |
| Ji SG 2017 | 30 | 49 | 19 | 49 |
| Li M 2007 | 16 | 32 | 11 | 32 |
| Li XQ 2009 | 16 | 30 | 5 | 32 |
| Miao CL 2007 | 37 | 43 | 30 | 44 |
| Yang XF 2006 | 22 | 30 | 16 | 30 |
| Yao SL 2004 | 16 | 24 | 10 | 22 |
| Yu HY 2012 | 24 | 32 | 16 | 32 |
| Zhang W 2011 | 21 | 46 | 7 | 30 |

**Table S4 KPS in two groups**

| Studies | Experimental | | Control | |
| --- | --- | --- | --- | --- |
|  | Events | Total | Events | Total |
| Qiao YX 2006 | 86.15 | 9.33 | 60 | 71.36 |
| Wang WR 2013 | 68.5 | 12.45 | 45 | 56.92 |

**Table S5**. Weight change in two groups

| Studies | Experimental | | Control | |
| --- | --- | --- | --- | --- |
|  | Events | Total | Events | Total |
| Bao WL 2011 | 15 | 45 | 14 | 48 |
| Li M 2007 | 26 | 32 | 17 | 32 |
| Li XQ 2009 | 10 | 30 | 9 | 32 |
| Qiao YX 2006 | 23 | 60 | 11 | 60 |
| Yu HY 2012 | 7 | 32 | 5 | 32 |

**Table S6. Pain relief in two groups**

| Studies | Experimental | | Control | |
| --- | --- | --- | --- | --- |
|  | Events | Total | Events | Total |
| Miao CL 2007 | 23 | 43 | 13 | 44 |
| Yang XF 2006 | 11 | 21 | 6 | 20 |
| Yu HY 2012 | 25 | 32 | 14 | 32 |

**Table S7. Myelosuppression in two groups**

| Studies | Experimental | | Control | |
| --- | --- | --- | --- | --- |
|  | Events | Total | Events | Total |
| He YZ 2016 | 3 | 42 | 11 | 50 |
| Wang WR 2013 | 4 | 45 | 6 | 45 |
| Yu HY 2012 | 11 | 32 | 20 | 32 |

**Table S8. Leukopenia in two groups**

| Studies | Experimental | | Control | |
| --- | --- | --- | --- | --- |
|  | Events | Total | Events | Total |
| Bao WL 2011 | 25 | 45 | 29 | 48 |
| Cao J 2016 | 11 | 40 | 20 | 40 |
| Duan HL 2018 | 2 | 30 | 15 | 30 |
| Huang ZF 2010 | 9 | 30 | 17 | 30 |
| Ji SG 2017 | 36 | 49 | 28 | 49 |
| Li XQ 2009 | 16 | 30 | 19 | 32 |
| Miao CL 2007 | 24 | 43 | 35 | 44 |
| Qi RF 2011 | 7 | 30 | 12 | 30 |
| Qiao YX 2006 | 11 | 60 | 50 | 60 |
| Sun J 2002 | 15 | 45 | 28 | 37 |
| Sun Y 2008 | 13 | 32 | 25 | 30 |
| Yang XF 2006 | 10 | 30 | 22 | 30 |
| Yao J 2018 | 23 | 100 | 21 | 100 |
| Yao SL 2004 | 7 | 24 | 12 | 22 |

**Table S9. Hemoglobin in two groups**

| Studies | Experimental | | Control | |
| --- | --- | --- | --- | --- |
|  | Events | Total | Events | Total |
| Bao WL 2011 | 16 | 45 | 20 | 48 |
| Ji SG 2017 | 37 | 49 | 28 | 49 |
| Li XQ 2009 | 11 | 30 | 13 | 32 |
| Qiao YX 2006 | 31 | 60 | 45 | 60 |
| Sun Y 2008 | 5 | 32 | 10 | 30 |

**Table S10. Thrombocytopenia in two groups**

| Studies | Experimental | | Control | |
| --- | --- | --- | --- | --- |
|  | Events | Total | Events | Total |
| Bao WL 2011 | 10 | 45 | 16 | 48 |
| Duan HL 2018 | 1 | 30 | 9 | 30 |
| Huang ZF 2010 | 7 | 30 | 13 | 30 |
| Ji SG 2017 | 21 | 49 | 15 | 49 |
| Li XQ 2009 | 6 | 30 | 10 | 32 |
| Qi RF 2011 | 2 | 30 | 6 | 30 |
| Qiao YX 2006 | 15 | 60 | 19 | 60 |
| Sun J 2002 | 11 | 45 | 24 | 37 |
| Sun Y 2008 | 2 | 32 | 3 | 30 |
| Yang XF 2006 | 7 | 30 | 19 | 30 |

**Table S11. Neutropenia in two groups**

| Studies | Experimental | | Control | |
| --- | --- | --- | --- | --- |
|  | Events | Total | Events | Total |
| Sun J 2002 | 13 | 45 | 26 | 37 |
| Yang XF 2006 | 8 | 30 | 20 | 30 |

**Table S12. Nausea and vomiting in two groups**

| Studies | Experimental | | Control | |
| --- | --- | --- | --- | --- |
|  | Events | Total | Events | Total |
| Bao WL 2011 | 21 | 45 | 31 | 48 |
| Cao J 2016 | 2 | 40 | 3 | 40 |
| Duan HL 2018 | 3 | 30 | 14 | 30 |
| Huang ZF 2010 | 14 | 30 | 26 | 30 |
| Li XQ 2009 | 17 | 30 | 21 | 32 |
| Qi RF 2011 | 4 | 30 | 7 | 30 |
| Sun J 2002 | 18 | 45 | 37 | 37 |
| Sun Y 2008 | 12 | 32 | 25 | 30 |
| Yang XF 2006 | 12 | 30 | 30 | 30 |

**Table S13. Constipation in two groups**

| Studies | Experimental | | Control | |
| --- | --- | --- | --- | --- |
|  | Events | Total | Events | Total |
| Bao WL 2011 | 7 | 45 | 8 | 48 |
| Li XQ 2009 | 4 | 30 | 5 | 32 |

**Table S14. Peripheral neurotoxicity in two groups**

| Studies | Experimental | | Control | |
| --- | --- | --- | --- | --- |
|  | Events | Total | Events | Total |
| Bao WL 2011 | 5 | 45 | 8 | 48 |
| Duan HL 2018 | 3 | 30 | 11 | 30 |
| Li XQ 2009 | 3 | 30 | 5 | 32 |

**Table S15. Alopecia in two groups**

| Studies | Experimental | | Control | |
| --- | --- | --- | --- | --- |
|  | Events | Total | Events | Total |
| Ji SG 2017 | 38 | 49 | 36 | 49 |
| Miao CL 2007 | 16 | 43 | 29 | 44 |
| Sun Y 2008 | 25 | 32 | 27 | 30 |
| Wang WR 2013 | 5 | 45 | 8 | 45 |
| Yao J 2018 | 25 | 100 | 24 | 100 |

**Table S16. Hepatotoxicity in two groups**

| Studies | Experimental | | Control | |
| --- | --- | --- | --- | --- |
|  | Events | Total | Events | Total |
| Bao WL 2011 | 2 | 45 | 2 | 48 |
| He YZ 2016 | 5 | 42 | 15 | 50 |
| Ji SG 2017 | 19 | 49 | 16 | 49 |
| Li XQ 2009 | 1 | 30 | 1 | 32 |
| Miao CL 2007 | 2 | 43 | 4 | 44 |
| Qi RF 2011 | 1 | 30 | 2 | 30 |
| Sun Y 2008 | 1 | 32 | 2 | 30 |
| Yao J 2018 | 5 | 100 | 6 | 100 |

**Table S17. Nephrotoxicity in two groups**

| Studies | Experimental | | Control | |
| --- | --- | --- | --- | --- |
|  | Events | Total | Events | Total |
| Bao WL 2011 | 0 | 45 | 1 | 48 |
| Ji SG 2017 | 2 | 49 | 1 | 49 |
| Li XQ 2009 | 0 | 30 | 1 | 32 |
| Miao CL 2007 | 1 | 43 | 4 | 44 |
| Qi RF 2011 | 1 | 30 | 1 | 30 |
| Sun Y 2008 | 0 | 32 | 1 | 30 |

**Table S18. Cardiotoxicity in two groups**

| Studies | Experimental | | Control | |
| --- | --- | --- | --- | --- |
|  | Events | Total | Events | Total |
| Qi RF 2011 | 1 | 30 | 2 | 30 |
| Sun Y 2008 | 1 | 32 | 1 | 30 |

**Table S19. Phlebitis in two groups**

| Studies | Experimental | | Control | |
| --- | --- | --- | --- | --- |
|  | Events | Total | Events | Total |
| Qi RF 2011 | 3 | 30 | 2 | 30 |
| Sun J 2002 | 2 | 45 | 0 | 37 |
| Sun Y 2008 | 13 | 32 | 11 | 30 |
| Yang XF 2006 | 1 | 30 | 0 | 30 |

**Table 20. Allergic in two groups**

| Studies | Experimental | | Control | |
| --- | --- | --- | --- | --- |
|  | Events | Total | Events | Total |
| Ji SG 2017 | 4 | 49 | 3 | 49 |
| Wang WR 2013 | 2 | 45 | 3 | 45 |
